# An Odontoid Fracture Causing Apnea, Cardiac Instability, and Quadriplegia

**DOI:** 10.1155/2012/821565

**Published:** 2012-09-12

**Authors:** Christian A. Bowers, Gregory F. Jost, Andrew T. Dailey

**Affiliations:** Department of Neurosurgery, Clinical Neurosciences Center, University of Utah, 175 N. Medical Drive East, Salt Lake City, UT 84132, USA

## Abstract

Odontoid fractures are typically associated with low rates of acute neurologic deficit and morbidity/mortality in nonelderly patients. In the patient in this case, traumatic injury triggered by a syncopal event led to a combined C1-C2 fracture and a fatal spinal cord injury with apnea, quadriplegia, and cardiovascular instability. We briefly review the anatomical basis for the pathophysiology of cardiac dysfunction following high-cervical spine injury and present an example of a worst-case scenario.

## 1. Introduction

C2 fractures account for 20% of acute cervical spine fractures. They are associated with low rates of acute neurologic deficit and mortality in younger patients (8.5% and 2.4%, resp.), whereas in the elderly (>65 years old) morbidity and mortality approach 50 and 10%, respectively [1, 2]. Type III odontoid fractures rarely cause neurologic deficit [3]; however, type III odontoid fractures make up 20% of C1-2 combination injuries and suggest more structural and mechanical injury than do isolated C1 or C2 injuries [3]. 

## 2. Case Presentation

**Figure 1:**
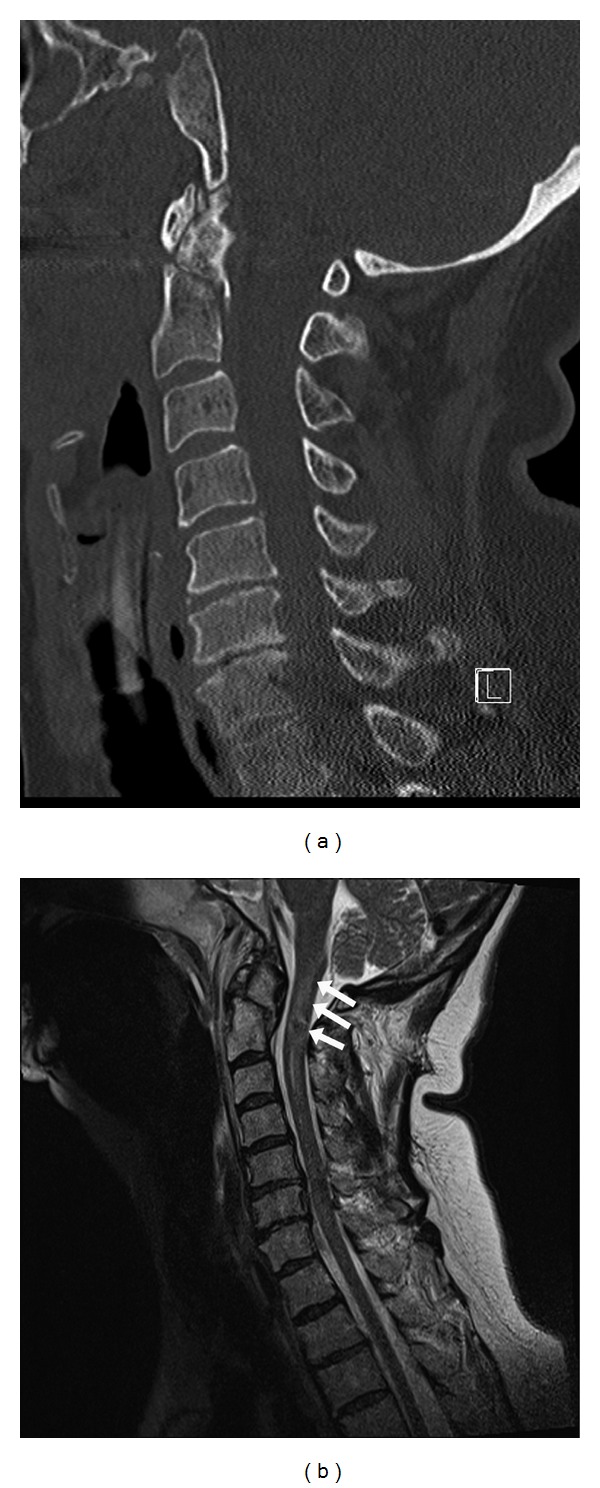
(a). Sagittal CT scan demonstrating type III odontoid fracture. (b). Sagittal nonenhanced T2-weighted MRI showing a cervical cord contusion with abnormal signal from the lower medulla to the inferior level of C3 (arrows).

A 58-year-old woman with a history of hypertension and cardiomyopathy fell onto her face while running to join her family in another room. The family heard her fall and found her with her neck hyperextended against a wall. The patient was not breathing, and the family immediately administered cardiopulmonary resuscitation. She was intubated and resuscitated from pulseless electrical arrest by the emergency medical services. Computed tomography imaging showed a type III odontoid fracture (Figure 1(a)) and a Jefferson-type fracture of the atlas (not shown). The patient had ST elevation on electrocardiogram and underwent emergent cardiac catheterization, which did not demonstrate an occluded vessel. It did show severe systolic heart failure with an ejection fraction of 15%, and the patient required multiple pressors to keep her heart beating. The patient awoke alert but quadriplegic. Magnetic resonance imaging of her cervical spine showed a cervical cord contusion behind C2 with abnormal signal from the medulla to the inferior C3 level (Figure 1(b)). She was fully dependent on a ventilator and required an external pacemaker because of repetitive bradyarrhythmias and asystolic pauses. Because of the low quality of life inherent in being a C2 quadriplegic with complete paralysis below the neck and lifelong ventilator dependence, the patient and family elected to withdraw care and the patient died. 

## 3. Discussion

The injury mechanism in this patient led to a combined C1-C2 fracture and a fatal spinal cord injury with apnea, quadriplegia, and cardiovascular instability. In fact, cardiovascular disease is a leading cause of death after spinal cord injury [4]. High spinal cord injuries interrupt the course of preganglionic sympathetic interneurons, which originate in the hypothalamus and exit the spinal cord from T1 to T6 to synapse with postganglionic sympathetic neurons in the middle cervical ganglion and stellate ganglion [4]. In contrast, the parasympathetic nervous system is less affected because it leaves the spinal cord at the level of the medulla oblongata and travels to the heart with the recurrent laryngeal and vagus nerve [4]. This shift towards parasympathetic-dominated heart control predisposes the patient to bradyarrhythmias and atrioventricular blocks [4]. It is possible that this patient experienced an arrhythmic event that caused her to fall, instead of tripping, and that this same arrhythmia was the cause of her subsequent cardiac dysfunction; however, multiple medical specialists believe that her heart failure and pacer dependence were a result of her traumatic contusion and spinal cord injury.
